# Data Management for Applications of Patient Reported Outcomes

**DOI:** 10.5334/egems.201

**Published:** 2018-05-10

**Authors:** E. A. Bayliss, H. A. Tabano, T. M. Gill, K. Anzuoni, M. Tai-Seale, H. G. Allore, D. A. Ganz, S. Dublin, A. L. Gruber-Baldini, A. L. Adams, K. M. Mazor

**Affiliations:** 1 Institute for Health Research, Kaiser Permanente Colorado, Denver, CO, US; 2 Department of Family Medicine, University of Colorado School of Medicine, Aurora, CO, US; 3 Section of Geriatrics, Department of Internal Medicine, Yale School of Medicine, New Haven, CT, US; 4 Meyers Primary Care Institute, a joint endeavor of the University of Massachusetts Medical School, Reliant Medical Group and Fallon Health, Worcester, MA, US; 5 Palo Alto Medical Foundation Research Institute, Palo Alto, CA, US; 6 Division of Geriatrics, Department of Medicine, David Geffen School of Medicine at UCLA, Los Angeles, CA, US; 7 Geriatric Research, Education and Clinical Center, VA Greater Los Angeles Healthcare System, Los Angeles, CA, US; 8 Kaiser Permanente Washington Health Research Institute, Seattle WA, US; 9 Division of Gerontology, Department of Epidemiology and Public Health, University of Maryland School of Medicine, Baltimore, MD, US; 10 Department of Research and Evaluation, Kaiser Permanente Southern California, Pasadena, CA, US

**Keywords:** patient reported outcomes, electronic health records, patient-centered care, data collection

## Abstract

**Context:**

Patient reported outcomes (PROs) are one means of systematically gathering meaningful subjective information for patient care, population health, and patient centered outcomes research. However, optimal data management for effective PRO applications is unclear.

**Case description:**

Delivery systems associated with the Health Care Systems Research Network (HCSRN) have implemented PRO data collection as part of the Medicare annual Health Risk Assessment (HRA). A questionnaire assessed data content, collection, storage, and extractability in HCSRN delivery systems.

**Findings:**

Responses were received from 15 (83.3 percent) of 18 sites. The proportion of Medicare beneficiaries completing an HRA ranged from less than 10 to 42 percent. Most sites collected core HRA elements and 10 collected information on additional domains such as social support. Measures for core domains varied across sites. Data were collected at and prior to visits. Modes included paper, clinician entry, patient portals, and interactive voice response. Data were stored in the electronic health record (EHR) in scanned documents, free text, and discrete fields, and in summary databases.

**Major themes:**

PRO implementation requires effectively collecting, storing, extracting, and applying patient-reported data. Standardizing PRO measures and storing data in extractable formats can facilitate multi-site uses for PRO data, while access to individual PROs in the EHR may be sufficient for use at the point of care.

**Conclusion:**

Collecting comparable PRO data elements, storing data in extractable fields, and collecting data from a higher proportion of eligible respondents represents an optimal approach to support multi-site applications of PRO information.

## Introduction

Creating a patient-centered model of care requires a cultural shift towards asking for, listening to, understanding, and acting on patients’ perspectives. Systematically integrating patient-reported outcomes (PROs) into healthcare delivery can contribute to this change. PROs, defined as “any report of the status of a patient’s health condition, health behavior, or experience with healthcare that comes directly from the patient, without interpretation…by a clinician or anyone else,” have been identified as a critical component of patient-centered care delivery [1]. Initially developed for research, PROs have long been used in clinical trials and epidemiologic studies to quantify constructs such as symptom burden, function, and quality of life, and have become the sine qua non of patient-centered outcomes research (PCOR) [2,3].

PROs can promote patient-centered care at two levels. At the individual level, PROs can inform treatment decisions and other care processes and guide shared decision making. At the aggregate level, PROs may serve as performance measures to guide quality improvement, health system management, and payment [4,5,6,7]. PROs are also essential outcomes for practice-based patient-centered research [8].

Most research on PROs has focused on psychometric properties of measures [9,10,11]. Additional studies have focused on targeting patients, data collection, processes for reporting results, and developing interventions for positive findings [12,13,14]. Based on this evidence and facilitated by improved technology, PROs have been enthusiastically endorsed by patients, clinicians, and policy makers [15,16]. However, relatively little is known about how and whether PRO use is likely to achieve its intended goals. Unknowns include how to minimize patient and clinician burden, optimize data collection and work flows, prioritize which PROs matter for most delivery of clinical care and for quality measurement/improvement, understand value to clinicians and patients, and apply aggregate data for performance measurement and population health [17,18]. In short, how to achieve, understand, and optimize PRO implementation.

**Figure 1:**
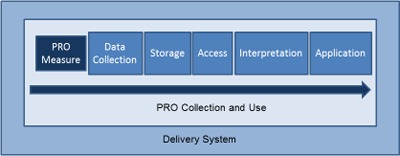
Process elements for systematic collection and use of PRO data. Underlined elements are examined through the Medicare Health Risk Assessment case study.

To systematically use PROs to inform care, it is important to understand how PRO data can be collected and stored, and whether systematic collection is likely to reach the respondent population of interest. If PRO data will be aggregated to assess quality or inform population health, it is additionally important to understand variation in measure selection across delivery settings. Using a case study of implementing the Medicare Health Risk Assessment in multiple care delivery systems, we examine the data management processes underlying systematic PRO collection and use. Figure 1 illustrates elements of systematically collecting and using PRO data.

## Case study: Medicare Health Risk Assessment

The Medicare Health Risk Assessment (HRA) is designed to identify patient-reported modifiable risk factors and health needs [19,20]. HRAs are a component of the Medicare Annual Wellness Visit which was implemented in 2011 as part of the Affordable Care Act. The HRAs are completed at or prior to the visit, and are addressed at the visit or as part of population care management. All Medicare Advantage and Medicare part B beneficiaries are eligible for Medicare Annual Wellness Visits and providers are reimbursed for these visits by the Centers for Medicare and Medicaid Services (CMS). As a result, HRA use is expanding rapidly. Although assessment of certain domains is required, the measures to assess these domains are not specified. HRA implementation provides an opportunity to better understand the spectrum of collecting, storing, and extracting PRO data.

The Health Care Systems Research Network (HCSRN) is comprised of research centers based in large community based integrated delivery systems that provide both clinical care and insurance coverage. Taken together, these health systems care for 16.8 million individuals, with over 75 percent of those individuals remaining continuously enrolled in the systems for at least one year. The systems are based in: Massachusetts, Pennsylvania, Maryland, Georgia, Michigan, Wisconsin, Minnesota, Texas, Colorado, California, Oregon, Washington, and Hawaii. Demographics of each system’s patient population mirrors the underlying geographic area. For example, the population cared for by Kaiser Permanente Mid-Atlantic is 35 percent African American; about 40 percent of the population cared for by Kaiser Permanente Southern California is of Hispanic ethnicity. Most systems are in medium to large urban areas, with the exception of Geisinger, in central Pennsylvania, and Marshfield Clinic, in central Wisconsin. Epic is the predominant electronic health record system used by HCSRN members, with two sites (Marshfield and Henry Ford) using mature in-house systems that have been in place for decades.

Each of these integrated delivery systems provides networked care and collects and stores clinical data in a common data model. Delivery systems associated with the HCSRN serve large populations of Medicare fee for service and Medicare Advantage beneficiaries across a range of service delivery models [21,22]. HCSRN delivery systems include both early and later adopters of HRA collection. Several store HRA data in extractable fields in electronic health records (EHRs) and aggregate data for population health in addition to using HRA data at the point of care. Health Risk Assessment implementation in these settings provided the opportunity to assess PRO data collection and storage across multiple delivery systems.

Using leaders of HCSRN research departments as points of contact, we sent a questionnaire inquiring about HRA content, data collection, data storage, the proportion of Medicare beneficiaries completing HRAs, and the dates at which HRA data collection became operational to 18 HCSRN delivery systems. Research department representatives collected responses from a range of operational and clinical leaders responsible for HRA implementation and use at each site. Thus responses reflect site-specific information available to these clinical and operational leaders.

The project was reviewed by the Kaiser Permanente Colorado Institutional Review Board and was determined not to be Human Subjects Research.

## Findings

Of 18 sites contacted, 15 (83.3 percent) sites provided some information on HRA content, collection, and data storage. Not all sites answered all questions, and some sites provided more than one response per question. The proportion of Medicare beneficiaries completing an HRA ranged from less than 10 to 42 percent. Two sites reported data collection since 2011, 6 sites since 2012, and 4 since 2013 or 2014. Data on collection dates were missing for 3 sites and data on proportion completed missing for 7 sites.

Content: All 15 sites reported collecting information on fall risk, mood, and functional status. Fourteen additionally assessed pain and advance directives; and 10 also assessed self-rated health and cognition. For no domain did all sites use the same PRO measure, although all sites used some variation of activities of daily living to assess function. Table 1 lists measures reported for the core HRA domains. Ten sites used the HRA data collection as an opportunity to inquire about other health-related issues. These included hearing and vision, incontinence, oral health, weight loss, sleep, food insecurity, alcohol and tobacco use, social support, physical living arrangements, sexual activity, and personal goals for self-management. (Table 2).

**Table 1:** Measures used to assess core Health Risk Assessment domains (N = 16 sites).

Domain	Measures (number of sites*)

**Self-Rated Health**	SF-36 (8) [23] WHO self-rated health (1) [24] “How would you describe your general health?” (1)
**Functional Status**	Activities of Daily Living (ADL) (10) Instrumental Activities of Daily Living (IADL) (5) Items from SF-12 (5) [25]
**Mood**	PROMIS Global 10-item scale (3) [26] PHQ-2 (5) [27] PHQ-9 (4) [28] GAD-2 (1) [29]
**Pain**	Item from PROMIS Global 10-item scale (10) [26] Brief Pain Inventory (1) [30]
**Cognition**	Mini-Cog (1) [31] AD-8 (1) [32] Mini Mental Status Exam (MMSE) (2) [33] “In the last year, have you or any of your friends and/or family felt concerned about changes in your memory?” (6)
**Fall Risk**	CDC 3-question screen (2) [34] “Have you fallen in the last 12 months or are you afraid of falling?” (1) “A fall is when your body goes to the ground without being pushed. Did you fall in the past 12 months?” (5)

* Not all sites reported specific measures and some sites reported using more than one measure. Abbreviations: SF-36: Short Form 36; WHO: World Health Organization; SF-12: Short Form 12; PROMIS: Patient Reported Outcomes Measurement Information System; PHQ: Patient Health Questionnaire; GAD: General Anxiety Disorder; CDC: Centers for Disease Control.

**Table 2:** Additional domains assessed across sites through Health Risk Assessment.

Domain	Number of sites

Alcohol and tobacco use	8
Physical activity	8
Seat belt use	7
Urinary incontinence	6
Hearing	6
Sleep quality	6
Weight loss or nutrition	6
Food insecurity	5
Home conditions (stairs, railings, tub bars, fire alarms	5
Sexual activity	5
Oral health	5
Vision	4
Social isolation/loneliness	4
Demographic information	3
Social support	3
Self-management goals/primary concern	2
Living arrangement	2
Past year medical care needs	2
Medication adherence	1
Aspirin use	1

Data collection methods: Sites used varying (and often multiple) approaches to engage patients for HRA completion: All sites except one collected HRA data at the point of care, 6 additionally collected data prior to the visit, and one site only collected data prior to the visit. Modes of administration included paper (10 sites), patient portal (5 sites), staff entry into the EHR (10 sites), tablet collection (4 sites), telephone (2 sites), and interactive voice response (4 sites). Five sites used single modes of administration — four with staff entry of data into the EHR and one using paper administration through the mail. The most modalities used by any site was five. Two sites used a combination of paper, interactive voice response, staff entry of information, an interactive patient portal, and tablets at the point of care.

Data storage: The multiple mechanisms for HRA data collection resulted in different approaches to data storage – often within the same site. Four sites reported scanning documents into the EHR, 9 reported storing data in discrete fields in the EHR, and 9 used text fields accessible in the EHR, but not readily extractable into other databases. Nine sites stored data in summary tables or other databases—potentially available for performance measurement, population health, and/or research. Information on HRA data collection and storage is summarized in Table 3.

**Table 3:** Health Risk Assessment data collection and storage by site.

Site	Estimated proportion of Medicare beneficiaries completing HRA in 2014	Timing of HRA completion		Mode of administration						Data storage			

		Point of care	Prior to visit	Paper	Patient portal questionnaire	Staff entry in EHR	Tablet	Telephone	IVR	Scanned document	Free text in EHR	Discrete fields in EHR	Summary tables or data warehouse

1	11%	x		x		x	x			x	x	x	x
2	20%	x	x	x	x		x		x	x		x	x
3	NR	x		NR						NR			
4	10%	x	x	x	x	x	x		x		x	x	x
5	17%	x	x	x	x	x	x		x		x	x	x
6	NR	x	x	x		x		x		x		x	
7	NR	x		NR							x	x	x
8	36%	x				x						x	
9	5%		x	x									x
10	43%	x	x	x	x	x		x	x	x		x	x
11	<10%	x				x					x	x	x
12	NR	x				x					x		
13	NR	x				x					x		
14	NR	x		x		x				x	x		
15	17%	x	x	x	x					x	x	x	x

* Abbreviations: HRA- Health Risk Assessment; IVR—Interactive Voice Response; EHR—Electronic Health Record; NR—Not Reported.

## Discussion

This case study of large scale, systematic PRO collection in integrated healthcare delivery systems illustrates a spectrum of approaches to selecting, collecting and storing PRO data. Fully realizing the potential of PROs to enhance patient centered care will require optimizing PRO selection and data management for multiple applications including use at the point of care, population health management, patient-centered outcomes research, and performance measurement within or across sites.

We found substantial variation in measure selection across sites. While CMS prescribes core domains to be included in HRAs, it does not prescribe specific measures, and sites selected different PRO measures to assess these domains. Using varied measures is sufficient for internal use of PRO data either at the point of care or for population health. Responses can prompt referrals for needed care, facilitate shared decision making, or lead to other clinical actions; and can be combined to manage population health or conduct research within a single site. Combining PRO data across sites for broader assessments of population health or research is more likely to be effective if domains are assessed with the same PRO measures. Using the same measures for function, fall risk assessment, cognition, and other domains at multiple sites would permit accurate comparisons of the domains of interest across populations, including assessing changes over time.

Using responses at the point of care requires that the information be available to clinicians for individual decision-making. While some sites reported multidimensional approaches to data collection and storage that resulted in easily visible PRO data, others reported more limited collection and access only to scanned documents. Formatted data in the EHR are usually easiest for clinicians to access and can facilitate use at the point of care. Scanned documents can also be accessed at the point of care; however, scanned documents preclude viewing longitudinal trajectories of PRO data even at the individual level. In comparison, aggregating PRO data for population health, possible performance measurement, and PCOR requires that responses be stored in readily extractable fields in the EHR. Collecting comparable PRO data elements and storing data in extractable fields represent an optimal combination for multi-site applications of PRO information.

Our case study had several limitations. We did not fully assess HRA implementation including how characteristics of the care delivery setting affect data management capacity [35]. As CMS provides financial incentives for the annual wellness visit, our findings reflect PRO implementation in response to external incentives rather than internal needs [36]. We focused on data collection and use and did not examine clinician attitudes towards using these PROs at the point of care or patients’ perceptions of response burden or usefulness. There is currently little evidence on the value and usefulness of PROs to clinicians. While many are supportive, others are wary of management burden and unintended consequences [7,37]. Information on both patients’ and clinicians’ perceptions of these or any other PRO is essential for making decisions about implementing PRO at scale.

Systematic PRO collection provides a potentially valuable resource for PCOR and longitudinal assessments of population health. For example, two national efforts (Sweden and the United Kingdom) are using pre- and post-operative PRO collection to guide internal quality improvement [1,38,39]. Using HRA data on a population level will require data collection from a larger proportion of the population (to ensure respondents are representative) and further study to understand the characteristics of respondents. In our assessment, the site with the highest HRA response rate only reported 42 percent of beneficiaries completing the HRA, and some sites reported less than 10 percent response rates. Having such a low response rate raises concerns that those who respond are not representative of the broader population of older adults. For instance, respondents might be considerably healthier (or sicker) on average than those who did not respond. Relatively little evidence exists on whether the reach and representativeness of PRO collection in general is adequate to support PRO use for performance measurement, PCOR and population health [1,2,39,40,41,42]. Target populations most likely to benefit from PRO use include medically and socially complex patients, and individuals for whom repeated measures of function help gauge recovery [43,44,45]. The National Quality Forum and others have called for research on best practices in this area [1,46,47,48].

## Conclusions

Providing sustainable patient-centered care at scale will require a cultural and process shift to measuring and acting on outcomes that matter to patients such as function and symptom burden [16,44]. If effectively implemented, systematically-collected and representative PRO data can help clinicians collaborate with patients; help care leaders, policy makers, and the public monitor interventions; and help researchers focus on outcomes that matter to patients. Based on the case of HRA collection within the HCSRN, effective PRO use at scale will require changes including more widespread use of standardized measures, effective and efficient modes of collection, and accessible and readily extractable data that facilitate PRO use on the population level, as well as for providing clinical care to individual patients.

## Author Information

Dr. Gill is the recipient of an Academic Leadership Award (K07AG043587) from the National Institute on Aging. Drs. Gill and Allore are supported by the Yale Claude D. Pepper Older Americans Independence Center (P30AG21342). Dr. Gruber-Baldini is supported by the University of Maryland Claude D. Pepper Older Americans Independence Center (P30 AG028747).
